# High-fat diet increases pain behaviors in rats with or without obesity

**DOI:** 10.1038/s41598-017-10458-z

**Published:** 2017-09-04

**Authors:** Zongbin Song, Wenrui Xie, Sisi Chen, Judith A. Strong, Mason S. Print, Joy I. Wang, Aleeya F. Shareef, Yvonne M. Ulrich-Lai, Jun-Ming Zhang

**Affiliations:** 1 Department of Anesthesiology, University of Cincinnati College of Medicine, 231 Albert Sabin Way, Cincinnati, OH 45267-0531 USA; 2 Department of Psychiatry and Behavioral Neuroscience, University of Cincinnati College of Medicine, 2170 East Galbraith Road, ML 0506, Metabolic Diseases Institute, Reading Campus, Cincinnati, OH 45237-0506 USA; 3 Department of Anesthesiology, Xiangya Hospital, Central South University, 87 Xiangya Road, Kaifu qu, Changsha, Hunan Province 410008 China

## Abstract

Obesity is associated with increased risk for chronic pain. Basic mechanisms for this association are poorly understood. Using a milder version of a radicular pain model, local inflammation of the dorsal root ganglion (DRG), we observed marked increases in mechanical and cold allodynia in rats of both sexes that were maintained on a high-fat diet (HFD) for 6 weeks prior to DRG inflammation. Notably, this increase in pain-related behaviors occurred in both Long-Evans and Sprague-Dawley rats despite the fact that the 6-week HFD exposure induced obesity (e.g., increased insulin, leptin, weight, and percent body fat) in the Long-Evans, but not Sprague-Dawley, strains. This suggested that HFD, rather than obesity per se, increased pain behaviors. Increased pain behaviors were observed even after a much shorter (1 week) exposure to the HFD but the effect was smaller. HFD also increased behavioral responses and paw swelling to paw injection of complete Freund’s adjuvant, a model of peripheral inflammatory pain. No change was detected in plasma cytokine levels in HFD rats. However, increased macrophage infiltration of the DRG was observed in response to the HFD, absent any pain model. The results suggest that HFD can increase pain even when it does not cause obesity.

## Introduction

Obesity is a rapidly growing global public health problem that increases the risk for many diseases^1^. The percentage of adults who are obese has increased sharply over the past decades, with developing countries leading the trend. For example, in the United States, ~30% of adults are obese and another ~30% are overweight^2^. Obesity-related diseases that have received much attention include diabetes, cardiovascular disease, and cancer^3^. However, human studies also show associations between obesity and chronic pain conditions such as fibromyalgia, arthritis, and headache^4–6^. Obesity is also associated with disk degeneration and low back pain, possibly due not simply to mechanical factors (e.g. increased lumbar spine load), but to adipokines and cytokines released from white fat^7, 8^.

Obesity induces chronic low grade inflammation and innate immune system activation^6, 9^. Obese adipose tissue contains more immune cells, including M1-polarized pro-inflammatory macrophages^10^. Leptin, an important metabolic signaling hormone released by adipocytes, is also pro-inflammatory^11^. Neurons in the sensory ganglia and spinal cord have leptin receptors, and leptin can enhance pain behaviors when injected intrathecally (i.t)^12^. Other pro-inflammatory cytokines shown to be locally or systemically elevated in obesity include interleukin-6 (IL-6), tumor necrosis factor α (TNF-α), and IL-1β^9, 10^, which may be released by the excess adipose macrophages^7^. Thus, obesity-induced inflammation may modulate pain.

Many animal studies on the relationship between pain and obesity used genetic models of obesity, e.g. Zucker rats and mice lacking leptin or its receptor^13–17^. In these animals, extreme obesity occurs due to lack of leptin feedback signaling from fat stores to brain regions that limit food intake and increase energy expenditure^18^. However, few cases of human obesity are due to monogenetic causes such as loss of leptin or its receptor^19, 20^. Instead, the rapid increase in obesity prevalence is thought to be due primarily to environmental factors, especially decreased physical activity and increased consumption of calorically-dense foods, with particular emphasis on dietary saturated fat^3, 21, 22^. Thus, studying obesity-pain interactions in animal models of high-fat diet-induced obesity arguably has greater translational relevance. Some preclinical studies show that diet-induced obesity increases arthritis severity in animal models, although most of these did not directly measure pain responses^23–26^. Enhanced pain responses in peripheral inflammation models in rodents with diet-induced obesity have been shown in a few studies^27–29^. However, to our knowledge there are no preclinical studies of obesity in radicular pain models.

This study aimed to examine effects of diet-induced obesity on pain, focusing primarily on our model of radicular pain in which local inflammation of the DRG is induced by inflaming the L5 lumbar DRG^30^. This mimics the local inflammation that occurs with ruptured disks without nerve compression. We used the model of diet-induced obesity first described by Woods *et al*., who showed that feeding Long-Evans rats *ad libitum* a semi-synthetic defined high-fat diet leads to increased weight, percent body fat and plasma leptin, and insulin resistance, in both sexes^31^. We used both Long-Evans rats, as used in the original obesity model, and Sprague-Dawley rats, as used in the original radicular pain model. Because Sprague-Dawley rats did not develop diet-induced obesity in our experiments, we were able to observe effects of the diet on pain sensitivity in the absence of elevated body weight or adiposity.

## Methods

All surgical procedures and the experimental protocol were approved by the institutional animal care and use committee of the University of Cincinnati and were in accordance with the National Institutes of Health Guide for the Care and Use of Laboratory Animals.

### Animals

Long-Evans and Sprague-Dawley rats (Harlan/Envigo, Indianapolis, IN) of both sexes were used, as indicated. Initially, we confirmed that the effect of diet on the behavioral response to the DRG inflammation model was grossly similar in females and males, and thereafter used males for most subsequent experiments as indicated. The study was not designed to investigate sex differences in detail. Rats were housed two per cage at 22 ± 0.5 °C under a cycle of 14-h light and 10-h dark. Sprague-Dawley rats were not specifically selected to be from either the obesity-prone or obesity-resistant sub strains^32^ and we did not observe a subset that was obesity-prone.

### High-fat diet

**Figure 1:**
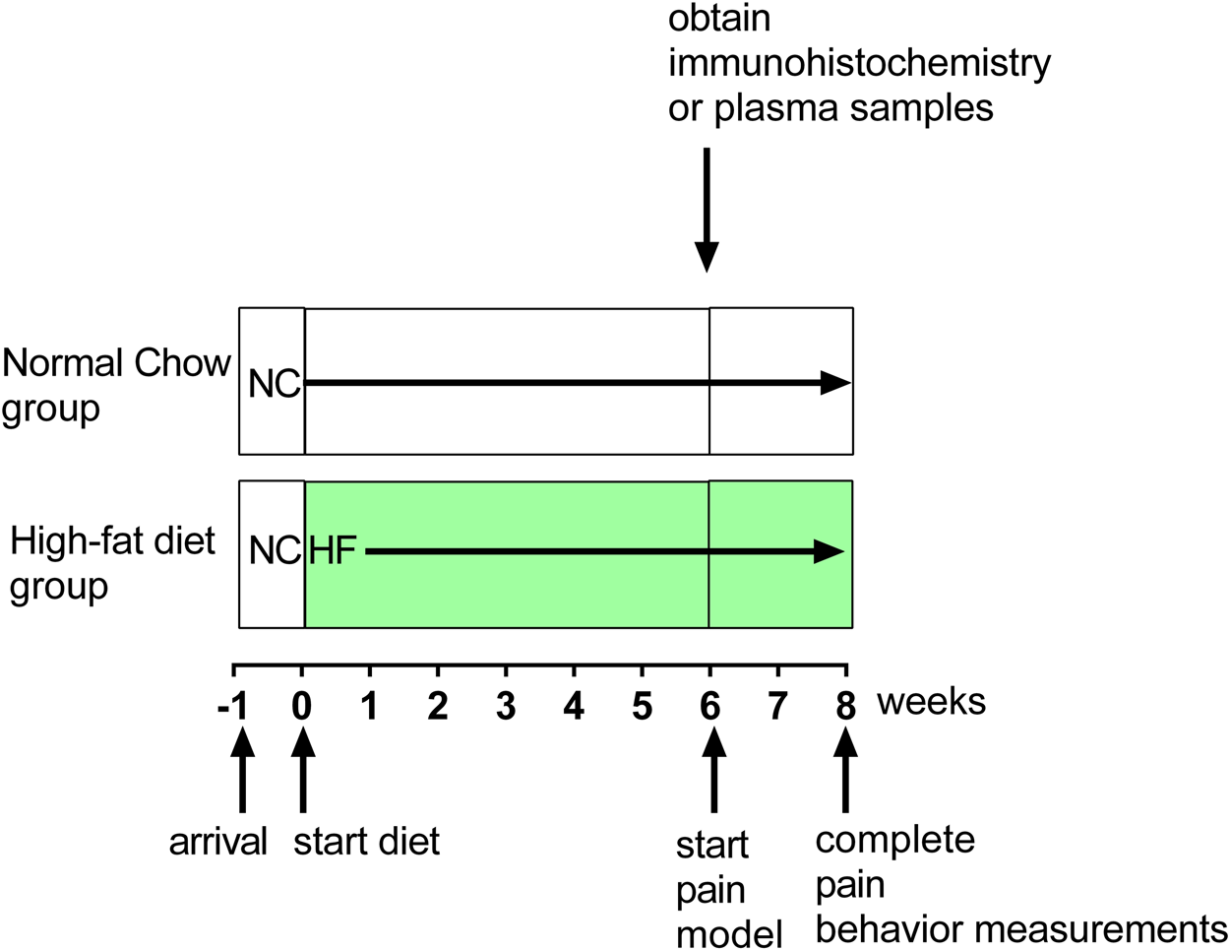
Experimental time course. For most experiments, animals weighing ~100 grams at arrival to the animal facility were acclimated to the facility for 4–7 days. Then, half the animals in each group were placed on the high-fat diet (“HF”) while the other half (size-matched rats with same arrival date) remained on normal chow (“NC”). For experiments measuring pain behaviors, the pain model was implemented after 6 weeks on the diet, and pain measurements continued for an additional 2 weeks while animals remained on the same diet they were on previously. For experiments measuring the effect of the diet on macrophage density or plasma cytokines, no pain model was implemented and samples were obtained after ~6 weeks on the diet, as indicated in the individual figure legends. Exceptions to this protocol are noted in the figure legends (Fig. 6).

All rats had *ad libitum* access to food and water. We used the paradigm of diet-induced obesity first described by Woods *et al*.^31^. The general experimental paradigm is shown in Fig. 1. Except where indicated, after acclimation to the animal facility, rats weighing ~100 grams were switched from the conventional open formula low fat chow (Harlan 7022, NIH-07 diet, 15% calories from fat) to a synthetic semi-purified diet with 40% of the calories from fat, primarily butter fat (Research Diets, catalog D03082706, “Tso diet”^31^). Control age-matched rats continued to receive the normal (low fat) chow. For behavior experiments, after 6 weeks on the diet, the DRG inflammation model of radicular pain or CFA model of peripheral inflammation was implemented. Animals continued on the same diet for two more weeks or as indicated. For experiments examining effects of diet on immunohistochemistry or cytokine/adipokines in the absence of a pain model, samples were obtained after ~6 weeks on the diet, corresponding to the time at which pain models were first implemented. Body composition (e.g., percent body fat). was assessed as follows: immediately after sacrifice, rat carcasses were individually sealed into air-tight plastic bags and stored at −20 °C; carcasses remained sealed in these bags during subsequent thawing, heating to 37 °C, and NMR measurement (Echo MRI, Echo Medical Systems, Houston, TX). Each carcass was measured twice and the values averaged. NMR was conducted only after all behavioral pain testing was complete due to concerns that the stress-associated with the NMR measurement (i.e., rats are essentially given a restraint stress during the NMR) could itself influence pain behavior and confound data interpretation.

### Surgical procedures for local inflammation of the DRG (LID)

The surgery was performed as previously described^33^ except that the dose of zymosan was lowered by a factor of 10 in order to make sure the pain behaviors in chow-fed rats did not approach maximum values (see Results). Briefly, under isoflurane anesthesia, an incision was made along the midline of the spine and the L5 intervertebral foramen was visualized by exposing L5 and L4 transverse processes. The immune activator zymosan (Sigma-Aldrich, catalog #Z4250, 10 µl of 0.2 mg/ml in incomplete Freund’s adjuvant) was injected beneath the L5 intervertebral foramen, above the DRG, via a needle bent at a 90-degree angle inserted into the intervertebral foramen.

### Procedure for localized paw inflammation with low volume Complete Freund’s Adjuvant (CFA)

CFA (Sigma-Aldrich, catalog F5881, containing 1 mg/mL of Mycobacterium tuberculosis, heat killed and dried in 85% paraffin oil and 15% mannide monooleate) was diluted with an equal volume of incomplete Freund’s adjuvant. Under brief isoflurane anesthesia, 10 µl of this mixture was injected subcutaneously (s.c.) into the heel region of one paw. As for the LID model, the CFA model was modified to be much milder, to avoid giving maximal pain behaviors in normal chow animals. For comparison, our previous study^34^ used a much larger (50 µL) volume of 50% CFA-50% artificial cerebrospinal fluid to induce paw inflammation. Hence, the dose used in the present study should not have had systemic effects; in other published studies using this model in rats, a dose of 150 µl CFA at 1 mg/ml, was shown to cause only local tissue and joint inflammation, with arthritic changes occurring only in the local joints and only at later times (30 days) than those measured in our experiments^35^, whereas higher doses (e.g. 5 mg/ml) were required to elicit systemic inflammation and arthritic changes in more distant and contralateral joints^36^.

### Paw measurements

At the time points indicated in supplemental Figs 3 and 4, under brief isoflurane anesthesia, the width and height of each hind paw were measured with calipers. The height (thickness) was measured by placing the one caliper on the top of the foot directly against the ankle, and the other caliper on the bottom of the foot at the heel. The width was measured at the point of widest medial-lateral distance, which was always near the heel. The two dimensions (average of 3 measurements each) were multiplied together to give a measure of paw cross-sectional area. Ipsilateral area was normalized to contralateral area to give an index of the amount of paw swelling.

### Behavior testing

Static mechanical allodynia (i.e., responses to punctate mechanical stimuli that are normally innocuous), was tested by applying a series of von Frey filaments to the heel region of the paws, using the up-and-down method^37^. A cutoff value of 15 grams was assigned to animals that did not respond to the highest filament strength used.

To measure dynamic mechanical allodynia (i.e., responses to light moving mechanical stimuli that are normally innocuous), a fine wisp of cotton was stroked mediolaterally across the plantar surface of the hindpaws to score the presence or absence of a brisk withdrawal response. This stimulus does not evoke a response in normal animals.

Cold sensitivity (cold allodynia) was scored as withdrawal responses to a drop of acetone applied to the ventral surface of the hind paw.

When observed, responses to acetone or light brush strokes consisted of several rapid flicks of the paw and/or licking and shaking of the paw; walking movements were not scored as positive responses.

Spontaneous guarding behavior was scored^38^ as 0 (no guarding, paw flat on floor), 1 (mild shift of weight away from paw), 2 (unequal weight bearing and some part of the foot not touching the floor), or 3 (foot totally raised or not bearing any weight); these scores were recorded just before each application of the von Frey filament (6 per paw total) and averaged. It was not feasible to blind the tester to the obesity/lean status of the animals. Previously we have found no difference between blinded and unblinded measurements using these pain models and behavior tests.

### Measurement of plasma cytokine and adipokine levels

Tail blood samples were obtained from awake rats after 45 days on the high-fat vs. normal chow diets, in the absence of any pain models, and collected into EDTA-containing tubes. Samples were briefly centrifuged to separate the plasma, which was stored at −80 °C until analysis. Samples were collected in the late afternoon (~3–4 hours prior to lights out) to maximize the ability to observe changes in leptin, which shows a circadian rhythm^39^. Plasma TNF-α, monocyte chemotactic protein 1 (MCP-1; systemic name CCL2), leptin, insulin, IL-6, and IL-1β were measured simultaneously from a single sample using the Millipore rat adipokine multiplex kit (catalog number RADPKMAG-80K) according to manufacturer’s protocol. A small volume from each sample was set aside for separate measurement of plasma adiponectin using the Millipore rat single plex assay (catalog number RADPNMAG-81K-01) according to manufacturer’s instructions, as this assay requires a higher sample dilution and uses a different buffer. For both kits, antibody-coupled beads were incubated with the plasma sample (antigen) after which they were incubated with biotinylated detection antibody before finally being incubated with the reporter molecule Streptavidin-PE conjugate. A broad sensitivity range of standards covering a ~4000-fold range was used to quantify over a wide range of cytokine concentrations. This captured immunoassay was then read by the Bio-Plex System which uses Luminex fluorescent-bead-based technology. This method has sensitivity and performance similar to ELISA methods, but requires much smaller sample volumes and is suitable for multiplexing^40^.

### Immunohistochemistry

Animals were first perfused with 0.1 M phosphate buffer until clear fluid was seen, followed by perfusion with 4% paraformaldehyde for 20 minutes. DRGs or spinal cord were post-fixed in 4% paraformaldehyde for one hour at room temperature then transferred to 0.1 M phosphate buffer, 4% sucrose overnight at 4 °C, then sections were cut at 40 µm on a cryostat. Macrophages in the DRG and microglia in the spinal cord were stained with an antibody to Iba-1 (Abcam, catalog ab5076, RRID AB_2224402, diluted 1:500). Activated satellite glia in the DRG and were examined using an antibody to glial fibrillary acid protein (GFAP; ImmunoStar catalog 22522, RRID AB_572240). Images from multiple sections of each DRG or spinal cord dorsal horn, selected at random without regard to the amount of signal observed, were captured using an Olympus BX61 fluorescent microscope with Slidebook 6.1 imaging acquisition software, and overall intensity (grey value of the signal above background) was measured and normalized by the area measured. In the DRG, only areas dominated by neuronal cell bodies were analyzed, rather than predominately axonal regions. Data from multiple sections was summarized as animal averages and the statistical analysis was applied to these average values.

### Statistics and data analysis

The general design of the experiments, except where indicated, was to obtain animals of ~100 grams from the vendor, acclimate them to the facility for several days, and then start half of the animals in each group (selected at random) on the high-fat diet. Each individual experiment (behavior, microscopy, obtaining plasma samples) was conducted concurrently with both high-fat diet animals and their normal chow fed controls. No animals were excluded from analysis. Sample sizes were based on our previous experience with the procedures used. Two-sided tests were used throughout. Graphpad Prism version 6 software was used for statistical analysis except where otherwise indicated. Behavioral, weight, and paw swelling time course data were analyzed using two-way repeated measures ANOVA with Sidak’s multiple comparisons posttest to determine on which days experimental groups differed. For these analyses, F values for the comparison between groups are given (as F_(degrees freedom in numerator, degrees freedom in denominator)_). In addition, for time course data, within each experimental group, one-way ANOVA with Dunnett’s posttest was used to determine on which day measurements differed from baseline. For these ANOVA analyses, F values for the comparison between baseline and all other time points are also given. Data for plasma adipokines and cytokines and leptin/adiponectin ratio were analyzed by 2-way ANOVA (comparing factors STRAIN and DIET), followed by Bonferroni’s post-hoc analysis, using GBStat analysis software. All cytokine/adipokine data presented were subjected to log transform prior to statistical analysis (either because ratios were being analyzed, or because the variance was not homogenous until being transformed, according to multiple tests; Hartley’s, Cochran’s, Levene’s, and Bartlett’s). None of the values for the samples presented were out of range. The particular statistical tests used for each variable are indicated in the respective text or figure legend. Where indicated, nonparametric tests were used as appropriate (based on D’Agostino & Pearson omnibus normality test or small sample size). Significance was ascribed for p < 0.05. Levels of significance are indicated by the number of symbols, e.g., *p = 0.01 to < 0.05; **p = 0.001 to 0.01; ***p < 0.001. Data are presented as mean ± S.E.M. except where indicated.

### Data availability

The data that support the findings of this study are available from the corresponding author upon reasonable request.

## Results

### High-fat diet consumption for 8 weeks affected body weight in Long-Evans but not Sprague-Dawley rats

**Figure 2:**
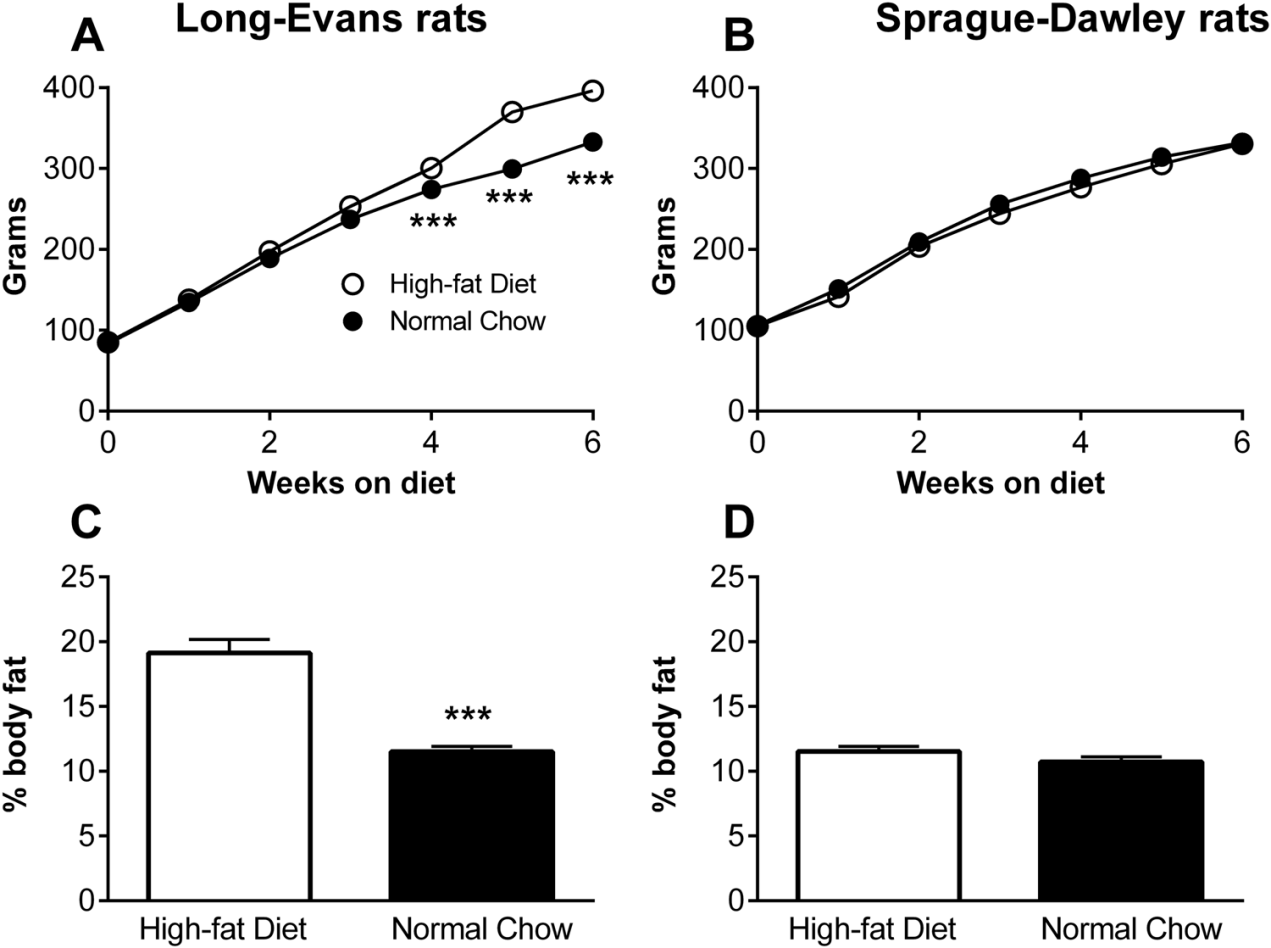
The effect of high-fat diet on body weight and adiposity differs between Long-Evans and Sprague-Dawley rats. Maintenance on a high-fat diet (vs. normal chow) increases the body weight of Long-Evans rats starting after 4 weeks of diet exposure (**A**), whereas this high-fat diet paradigm does not alter the body weight of Sprague-Dawley rats (**B**). Pain models were implemented at the 6 week time point in this study. ***p < 0.001, significant effect of diet at the indicated time points (2-way repeated measure ANOVA with Sidak’s multiple comparisons posttest; F_(1, 14)_ = 20.4 for **A** and 0.66 for **B**). N = 8 male rats per group. Similarly, percent body fat measured after the completion of pain behavioral testing (i.e., after 8 weeks of the indicated diet), was increased by the high-fat diet in Long-Evans rats (**C**) but not in Sprague-Dawley rats (**D**). ***p < 0.001, significant effect of diet (t-test). N = 8 male rats per group; half the animals in each group received the low dose DRG inflammation model after 6 weeks on the diet; significantly higher percent body fat in animals fed high-fat chow was observed in both subgroups of Long-Evans rats (with or without pain model; p < 0.01, t- tests), but in neither subgroup of Sprague-Dawley rats.

In this study, we used the paradigm of diet-induced obesity first described by Woods *et al*., who showed that *ad libitum* feeding of adult Long-Evans rats with a semi-synthetic defined high-fat diet for 10 weeks led to increased caloric intake, body weight, percent body fat and plasma leptin, as well as insulin resistance, in both males and females, and that the impact of high-fat diet on body weight emerged after just 3 weeks^31^. We examined both Long-Evans rats (because these were originally used in development of the diet-induced obesity model that we used), and Sprague-Dawley rats (because this strain was used in development of the pain models we used). We observed that *ad libitum* consumption of high-fat diet increased the body weight of Long-Evans rats, as previously reported^31^, and this increase preceded implementation of the pain model in our experimental design (Fig. 2). High-fat diet-fed Long-Evans rats also showed a significant increase in percent body fat measured by NMR after sacrifice (8 weeks on diet). However, high-fat diet consumption did not alter the body weight or adiposity (percent body fat) of Sprague-Dawley rats, consistent with the prior observation that longer durations of high-fat feeding are required to induce obesity and its associated metabolic disruptions in this rat strain^41^. Unlike some previous reports^42, 43^, we did not observe substantial inter-individual differences in susceptibility to high-fat diet-induced obesity within this strain. Taken together, these results suggest that this 8-week paradigm of high-fat diet induces obesity in a strain-dependent manner.

### Long-Evans rats maintained on a high-fat diet show larger responses to the milder LID model

**Figure 3:**
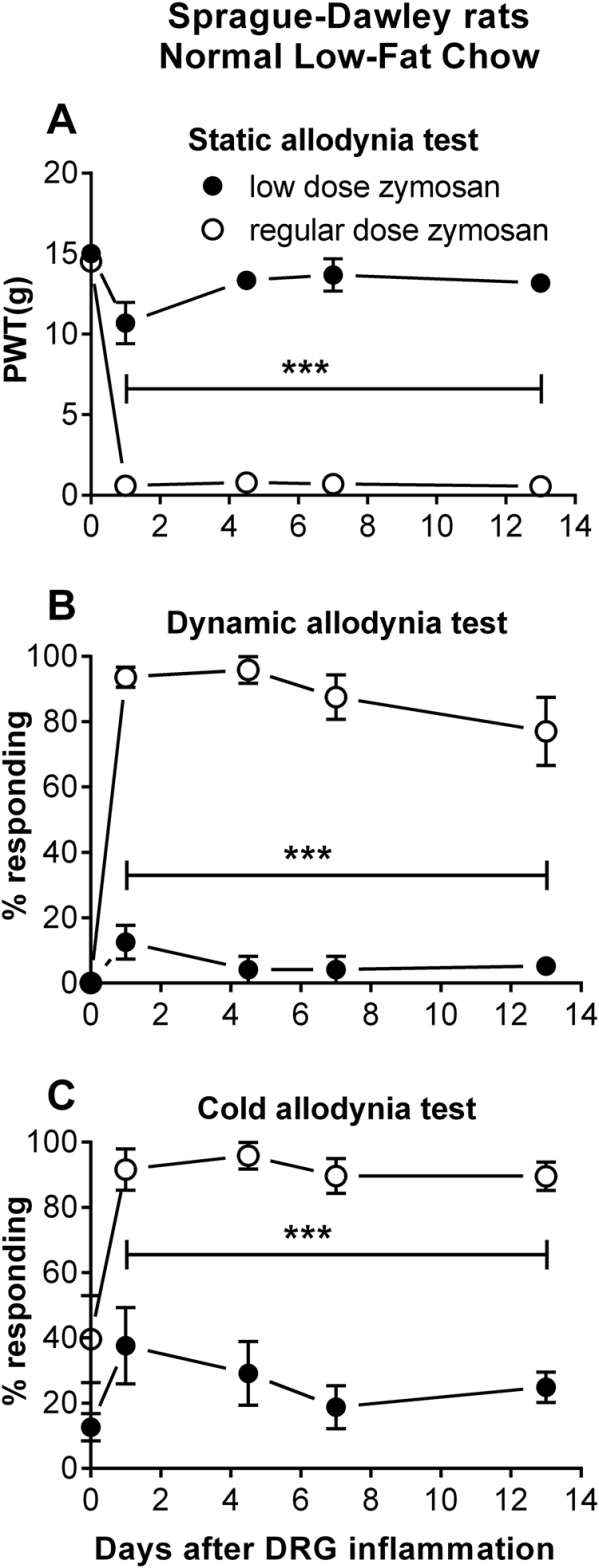
Low dose zymosan caused smaller, more transient pain behaviors in rats fed normal chow. Baseline measurements (average of 2) are plotted on postoperative day (POD) 0. On POD 0, the L5 DRG was injected with 10 µL incomplete Freund’s adjuvant (IFA) containing either 20 µg of zymosan (“regular dose zymosan”) as used in our previous studies, or 2 µg of zymosan (“low dose zymosan”). The regular dose caused a marked decrease in von Frey paw withdrawal threshold (PWT; **A**), and an increase in mechanical allodynia (withdrawal response to stroking with a cotton wisp; **B**) and cold allodynia (withdrawal response to a drop of acetone on the paw; **C**). These responses were significantly different from baseline starting on POD 1 and were maintained on all subsequent days of testing (one-way ANOVA with Dunnett’s posttest within the regular dose group; F_(5, 42)_ = 1032 for von Frey data, 30.2 for mechanical allodynia data, and 8.1 for acetone data). However, in the low dose group, the magnitude of the zymosan-induced pain behaviors was markedly attenuated, and differed significantly from baseline only in the von Frey test at POD 1 (one-way ANOVA with Dunnett’s posttest within the low dose group, F_(4, 35)_ = 3.9 for von Frey data, 1.5 for mechanical allodynia data, and 1.3 for acetone data). N = 8 Sprague-Dawley rats per group. Each group contains equal numbers of male and female rats; no obvious sex differences were observed so data from males and females have been combined. Data from the “regular dose zymosan” group are contemporaneous data taken from our recently-published study^31^. ***p < 0.001, significant different between the 2 groups at all post-baseline time points, 2-way repeated measure ANOVA with Sidak’s multiple comparison posttest; F_(1, 14)_ = 633.7 (**A**), 495.2 (**B**), and 91.9 (**C**).

**Figure 4:**
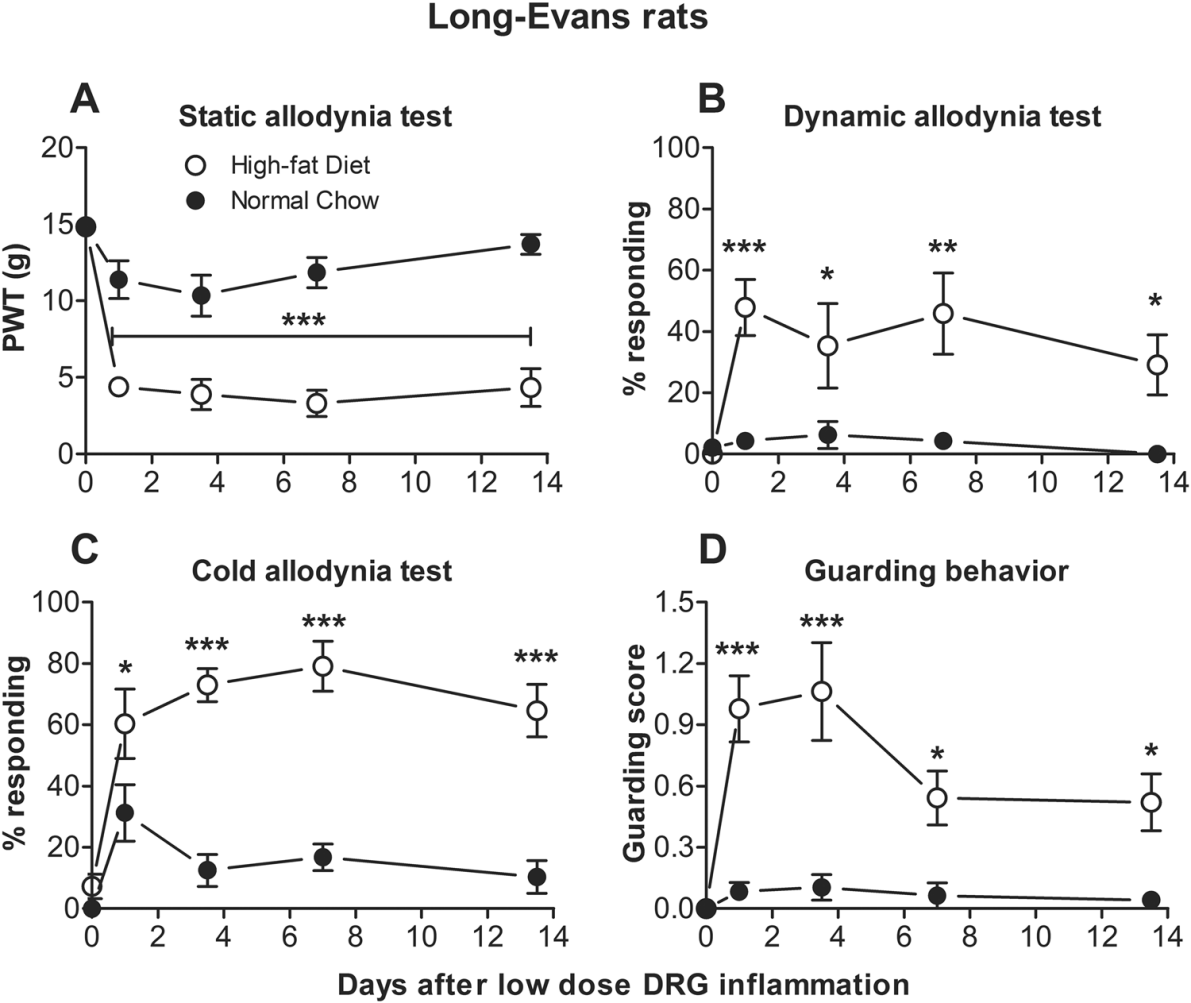
The low dose DRG inflammation model causes marked pain behaviors in Long-Evans rats maintained on a high-fat diet. The low dose DRG inflammation model was implemented on day 0, after animals had been maintained on the indicated diet for 6 weeks. Baseline behaviors were measured twice just before the pain model was implemented (average plotted on day 0). The high-fat diet group, but not the normal chow group, responded to the DRG inflammation with a marked decrease in von Frey threshold (**A**) and an increase in dynamic mechanical allodynia (**B**), cold allodynia (**C**), and guarding behavior (**D**). N = 8 Long-Evans rats per group. Each group contains equal numbers of male and female rats; data from males and females have been combined. Data for each sex plotted separately, including animal weights, can be viewed in supplemental Fig. 1. *p < 0.05; **p < 0.01; ***p < 0.001; significant difference between groups at indicated time points (2-way repeated measure ANOVA with Sidak’s multiple comparison posttest; F_(1, 14)_ = 48.9, von Frey data; 10.8, mechanical allodynia data; 67.0, acetone data; 42.5, guarding data).

The diet-induced obesity model that we used here was initially characterized in Long-Evans rats^31^, whereas Sprague-Dawley rats are initially relatively resistant to weight gain and metabolic disorders on this diet (see Discussion). Therefore, we first assessed the impact of diet on pain behaviors in the zymosan DRG inflammation model in Long-Evans rats. However, in this model as originally implemented, the behavior responses generally reached near maximal values, making it difficult to observe an increase in pain due to high-fat diet. To avoid these ceiling effects, in this and following experiments, we modified the zymosan model by lowering the dose from 20 µg/10 µl injection (used in our previous studies) to 2 µg/10 µl. As shown in Fig. 3, in animals maintained on the conventional low fat chow, low dose zymosan induced only mild, transient mechanical allodynia that lasted for one day as compared to near-maximal behavioral responses observed with the regular dose zymosan model. Using this milder model, we found that in Long-Evans rats maintained on a high-fat diet, mechanical and cold allodynia following low dose zymosan injection into the DRG were significantly greater than in normal chow-fed controls (Fig. 4). In this experiment we also measured guarding behavior, considered a sign of spontaneous pain^38^, and this was also significantly higher in the high-fat diet-fed rats fed at all post-surgical time points. Pain behaviors in normal chow-fed Long-Evans rats did not differ significantly from baseline on most post-operative days (one-way ANOVA with Dunnett’s posttest within the normal chow group; F_(4, 35)_ = 3.5 for von Frey data, significantly different from baseline on day 3 only; 0.73 for mechanically allodynia data; 4.1 for acetone data, significantly different from baseline on day 1 only; 0.76 for guarding data). The responses of Long-Evans males and females to the diet and to the low dose zymosan DRG inflammation were grossly similar, and data from both sexes have been combined in Fig. 4. The main exception was an apparently smaller mechanical allodynia response in female vs. male rats fed the high-fat diet. Data for males and females plotted separately can be seen in Supplemental Fig. 1.

### High-fat diet enhanced local DRG inflammation-induced pain behaviors in Sprague-Dawley rats in the absence of significant weight gain

**Figure 5:**
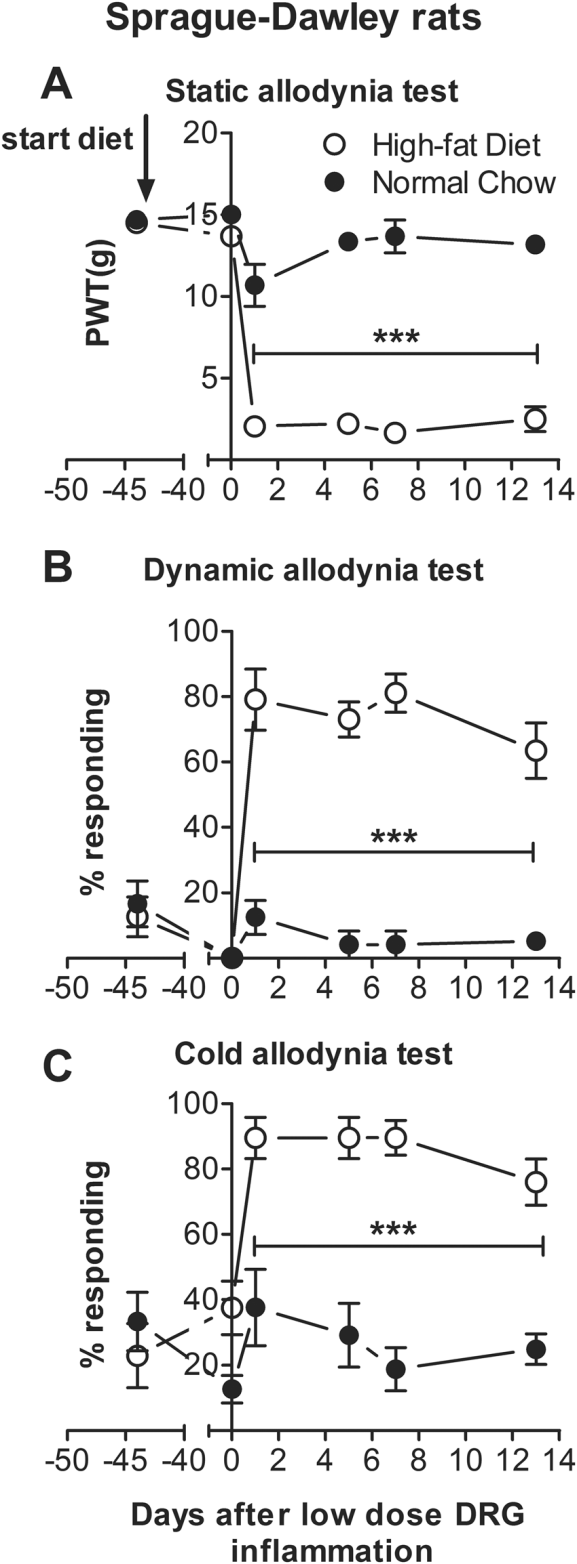
The low dose DRG inflammation model causes marked pain behaviors in Sprague-Dawley rats maintained on a high-fat diet. Baseline behaviors were measured just before animals were started on the diet (high-fat diet group; control animals continued on the standard low fat chow diet) (plotted on POD −44). Behaviors were measured again after 6 weeks on the diet and just prior to surgery for DRG inflammation with low dose zymosan (POD 0). The high-fat diet group, but not the normal (low fat) chow group, responded to the zymosan (2 µg) injection into the DRG with a marked decrease in von Frey threshold (**A**) and an increase in dynamic mechanical allodynia (**B**) and cold allodynia (**C**). N = 8 Sprague-Dawley rats per group (chow-fed rats, same data as those shown in Fig. 3). Each group contains equal numbers of male and female rats; no obvious sex differences were observed so data from males and females have been combined. Data for each sex plotted separately, including animal weights, can be viewed in supplemental Fig. 2. ***p < 0.001; significant difference between groups at indicated time points (2-way repeated measure ANOVA with Sidak’s multiple comparison posttest, F_(1, 14)_ = 379.7, von Frey data; 154.6, mechanical allodynia data; 63.9 acetone data).

We then tested pain behaviors in the Sprague-Dawley rats that did not show significant weight gain after being on the high-fat diet for 6 weeks. Similar to the Long-Evans rats, consumption of a high- fat diet for 6 weeks prior to DRG inflammation with low-dose zymosan markedly increased subsequent pain behaviors compared to rats maintained on normal (low fat) chow. As shown in Fig. 5, high-fat diet intake alone did not alter pain behaviors (as assessed on POD 0 just prior to DRG inflammation), although the measured values approach cut-off values and are not well-suited to detecting changes in baseline mechanical and cold thresholds. However, after DRG inflammation with the low dose zymosan, the animals fed the high-fat diet showed marked decreases in von Frey mechanical threshold and increases in cold and mechanical allodynia that were significantly different from the pre-surgery baseline on all post-surgery days tested (ANOVA with Dunnett’s posttest within that group; F_(4, 35)_ = 80.9 for von Frey data; 26.0 for mechanical allodynia data; 11.3 for acetone data), and that differed from the age- and sex-matched animals maintained on the low fat diet at all post-surgery time points (2 way repeated measures ANOVA with Sidak’s posttest). Interestingly, in the high-fat diet-fed rats, the magnitude of the measured behaviors was comparable to that observed in normal chow-fed rats at the higher zymosan dose (compare Figs 5 and 3). The responses of males and females to the diet and to the low dose zymosan DRG inflammation were grossly similar, and data from both sexes have been combined in Fig. 5. Data for males and females plotted separately can be seen in Supplemental Fig. 2.

### Long-Evans and Sprague-Dawley rats maintained on a high-fat diet also show larger responses to a peripheral inflammation pain model

Next, we tested the effect of the high-fat diet on another model of inflammatory pain – inflammation of the hindpaw by injection of CFA. In previous studies using the CFA model, in which we injected 50 µL of 50% CFA into the paw, the pain behaviors were near-maximal^34^, which would make it difficult to observe further enhancement of pain by the high-fat diet. We therefore modified the model by reducing the 50% CFA injection volume to 10 µl. As shown in Supplemental Fig. 3, this modification unmasked an exacerbating effect of high-fat diet on pain behaviors in Long-Evans rats, especially in the static allodynia (von Frey) test. For that test, the thresholds differed significantly between the 2 groups on all post-CFA days tested (two-way ANOVA with Sidak’s posttest); within the normal chow group the thresholds differed from baseline on only days 1 and 3, while within the high-fat diet group thresholds differed from baseline at all time points tested (one-way ANOVAs with Dunnett’s Multiple Comparison Posttest within each group; F_(4, 25)_ for the high-fat diet group and normal chow groups respectively = 45.0 and 4.6). For the dynamic allodynia and cold allodynia tests, the increased responding in the high-fat diet group reached significance only on post-CFA day 3, possibly due to large inter-individual variation within the high-fat diet group. Within-group analyses showed that the normal chow group did not differ from baseline on any day post-CFA for either of these two tests, whereas the high-fat diet group differed from baseline on post-CFA day 3 for the dynamic allodynia test and for post-CFA days 1 through 7 for the cold allodynia test (one-way ANOVAs with Dunnett’s Multiple Comparison Posttest within each group; F_(4, 25)_ for the high-fat diet group and normal chow groups respectively = 1.90 and 0.75 for mechanical allodynia test; and = 4.8 and 1.6 for the acetone test). The degree of paw swelling was also significantly larger in animals maintained on the high-fat diet, at all post-CFA time points. The within-group analyses indicated that the paw swelling index was significantly greater than baseline on all post-CFA days tested for both diet groups (one-way ANOVAs with Dunnett’s Multiple Comparison Posttest within each group; F_(4, 25)_ for the high-fat diet group and normal chow groups respectively = 45.2 and 10.5). Very similar results were obtained using Sprague-Dawley rats (Supplemental Fig. 4).

### A shorter exposure to high-fat diet was able to increase pain behaviors but the effect was short-lasting and less robust

**Figure 6:**
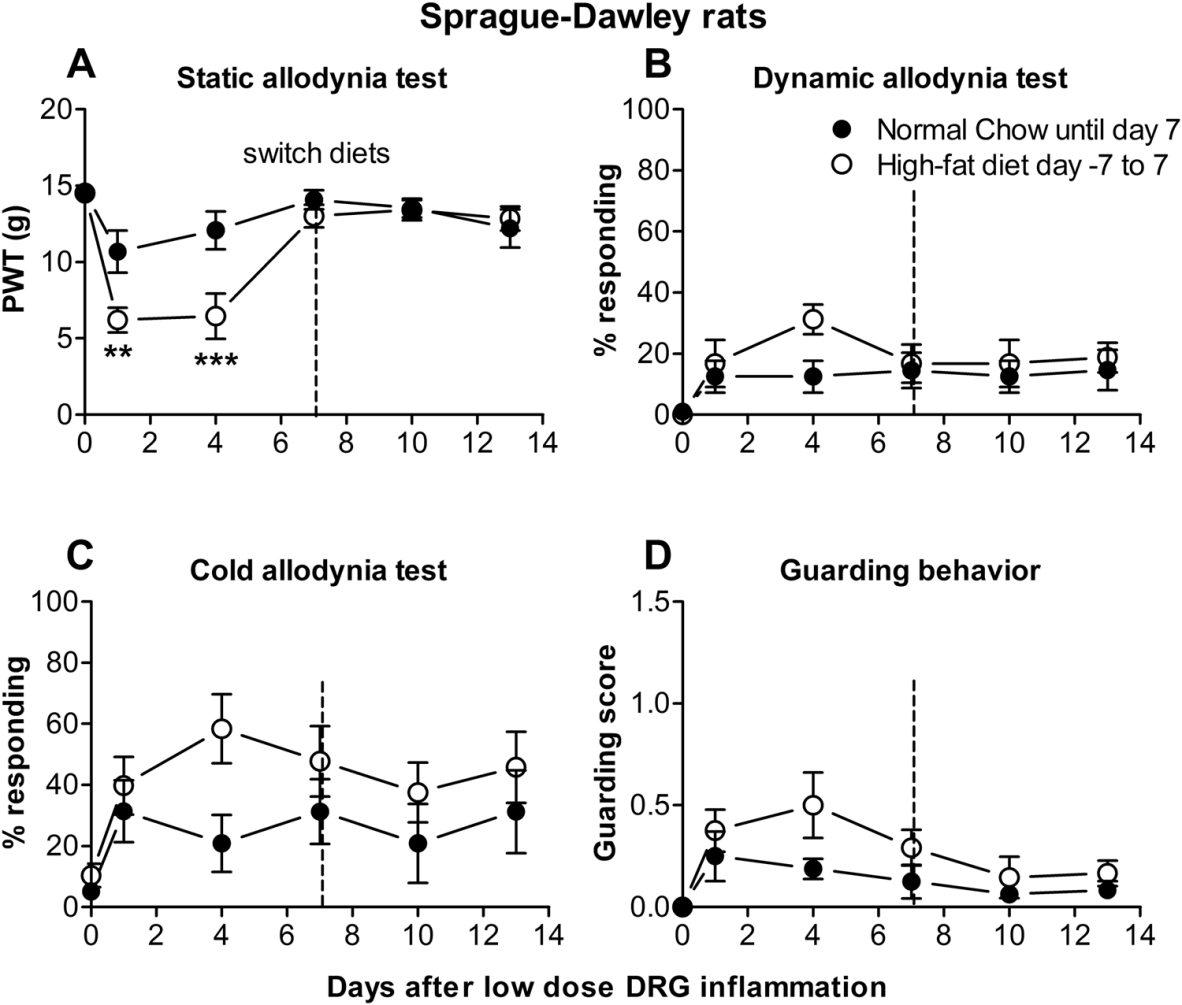
A shorter exposure to high-fat diet has a more modest effect on pain behaviors in the low dose DRG inflammation model. Sprague-Dawley rats were maintained on normal chow until reaching a weight of ~280 grams, to have age and weight comparable to animals in the previous experiments at the time the pain model was implemented. One week before the low dose DRG inflammation model was implemented, half the rats were switched to the high-fat diet, which was continued until one week after the pain model was implemented. After the behavior measurements on POD 7, the diets of the two groups were switched and behaviors measured for another 2 weeks. The short term (day −7 to 7) high-fat diet group responded to the DRG inflammation with a more modest decrease (compare to Fig. 5) in the von Frey threshold (**A**) that differed significantly from the normal chow group on POD 1 and 3. The groups did not significantly differ for the dynamic allodynia (**B**), cold allodynia (**C**), or guarding behavior (**D**) tests. For ease of comparison, graphs have the same y-axis scales as Figs 4 and 5. N = 8 male Sprague-Dawley rats per group. **p < 0.01; ***p < 0.001; significant difference between groups at indicated time points (2-way repeated measure ANOVA with Sidak’s multiple comparison posttest; F_(1,14)_ = 5.24 (**A**), 1.52 (**B**), 2.13 (**C**), 3.56 (**D**)).

The above findings suggested that diet per se, rather than obesity, might cause increased sensitivity in the pain models used. To examine this further, we tested the effects of feeding high-fat diet for only one week before implementing the low dose DRG inflammation pain model, instead of for 6 weeks as in all other experiments. The diet was continued for another 7 days after the model was implemented, at which time the diets were switched between the two groups. This experiment was done in Sprague- Dawley rats, which as shown above do not gain weight or increase body fat but do have an exacerbated pain phenotype following 6 weeks of high-fat diet intake. As shown in Fig. 6, a shorter duration of high-fat diet intake resulted in a less robust and shorter-lasting effect on pain behaviors; differences between the normal chow and short-term high-fat diet groups reached significance for only the von Frey test at 1–3 days post-surgery, and this effect resolved by day 7. Note that on days 1–3 the von Frey threshold was ~6 grams following the short-term high-fat diet group, compared to ~2 grams following longer term (6 week) high-fat diet intake (Fig. 5). Moreover, switching the normal chow group to high-fat diet at the 7 day time point did not activate pain behaviors. The short term diet exposures did not result in any significant weight differences between the groups (Supplemental Fig. 5). Preliminary data also showed that initiating high-fat diet consumption at 14 days after the onset of the CFA model did not activate pain behaviors (data not shown).

### High-fat diet consumption for 6 weeks affected plasma adipokines in Long-Evans but not in Sprague-Dawley rats

**Figure 7:**
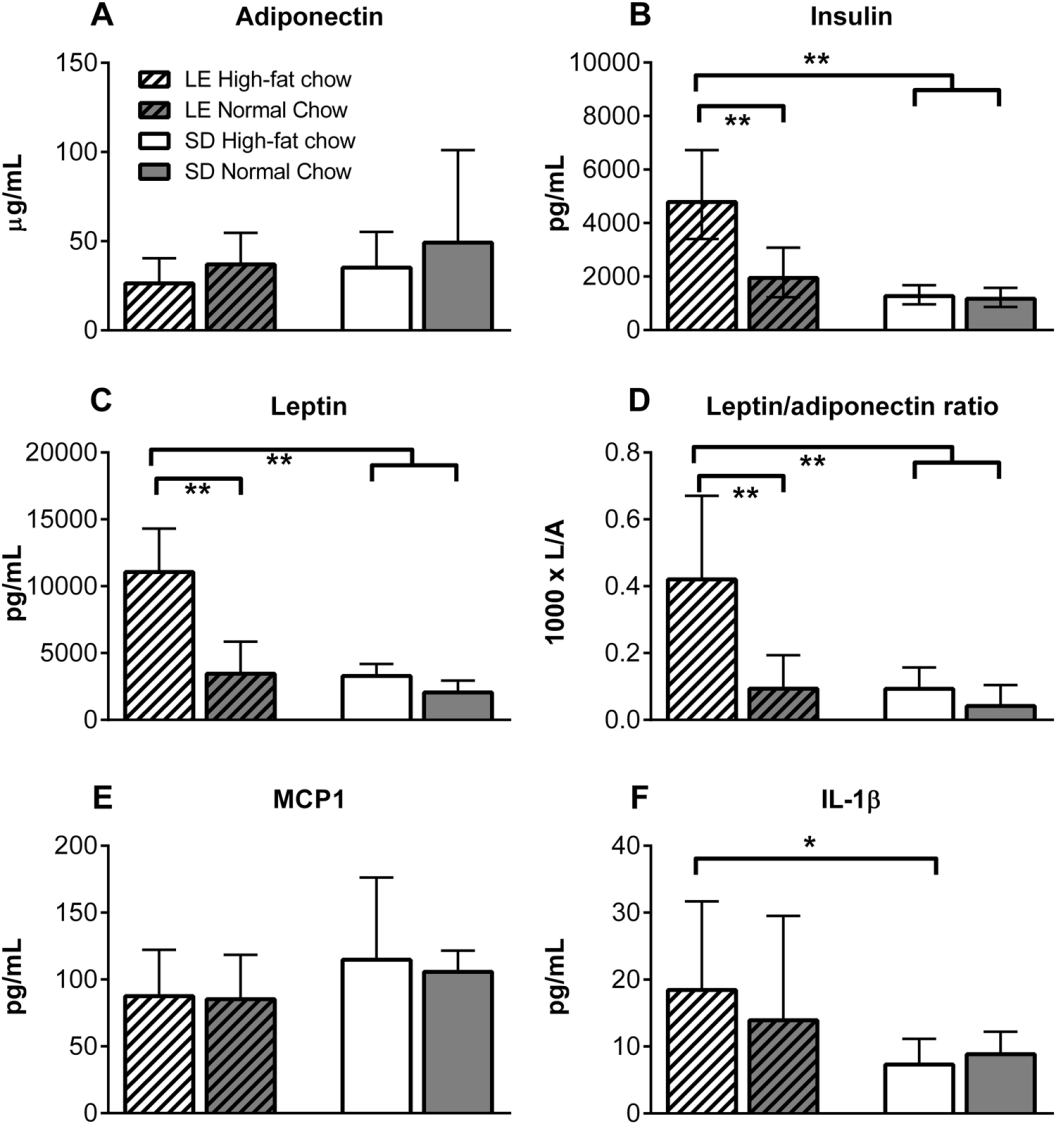
Effects of diet and strain on selected plasma adipokines and cytokines. Plasma samples were collected from tail blood at 45 days after rats were either placed on the high-fat diet or continued on the standard normal (low fat) chow. Samples were obtained before any pain models were implemented. Hatched bars indicate Long-Evans (LE) strain; open bars Sprague-Dawley (SD) strain. White bars indicate high-fat diet, gray bars indicate normal chow. Values shown are geometric means ± 95% confidence interval; statistical analysis was performed on log transformed data. High-fat diet increased plasma insulin (**B**) and leptin (**C**) only in the Long-Evans strain. Adiponectin levels were not significantly affected by strain or diet (**A**), but the ratio of leptin/adiponectin (L/A) was significantly elevated by high-fat diet only in Long-Evans rats (**D**). Plasma MCP did not differ between the groups (**E**). There was no effect of diet on plasma levels of IL-1β, but there was a significant effect of strain (**F**). *p < 0.05; **p < 0.01, significant difference between the indicated groups (two-way ANOVA with Bonferroni posttest comparing each group to the other 3 groups. The F values for the ANOVA analyses are shown in Supplemental Table 1. N = 8 male rats per group. At the time the sample was taken, the high-fat diet significantly increased weight in LE rats (438.5 ± 7.0 vs. 409.0 ± 8.3 grams, p < 0 0.05) but not in SD rats (357.5 ± 7.1 vs, 354.3 ± 11.9 grams) based on one-way ANOVA.

Some adipokines that are elevated in obesity are candidates for increasing inflammation and pain (e.g. leptin), but the observation that Sprague-Dawley rats also showed increased pain without becoming obese on the high-fat diet raised the question of whether this strain nonetheless had an altered adipokine profile as previously reported in Long-Evans rats. We examined plasma adipokines associated with the obese metabolic profile in rats of both strains after 6-weeks on the high-fat vs. normal chow diets, in the absence of any pain models. As shown in Fig. 7, insulin and leptin were elevated in Long-Evans rats, but not Sprague-Dawley rats, by the high-fat diet. High-fat diet did not alter plasma adiponectin in either strain, however the plasma leptin/adiponectin ratio was elevated by high-fat diet specifically in Long-Evans rats.

We also measured plasma cytokines that have been reported to be affected by diet in some studies. Neither MCP1 (Fig. 7E) nor IL1-β (Fig. 7F) was significantly affected by diet; however there was a significant strain effect on IL1- β, which was lower in Sprague-Dawley rats. IL-6 and TNF- α were also measured, however, for these analytes, many of the samples had cytokine levels that were below the limit of assay detection, precluding definitive interpretation of these data.

### High-fat diet increased macrophage density in the DRG

**Figure 8:**
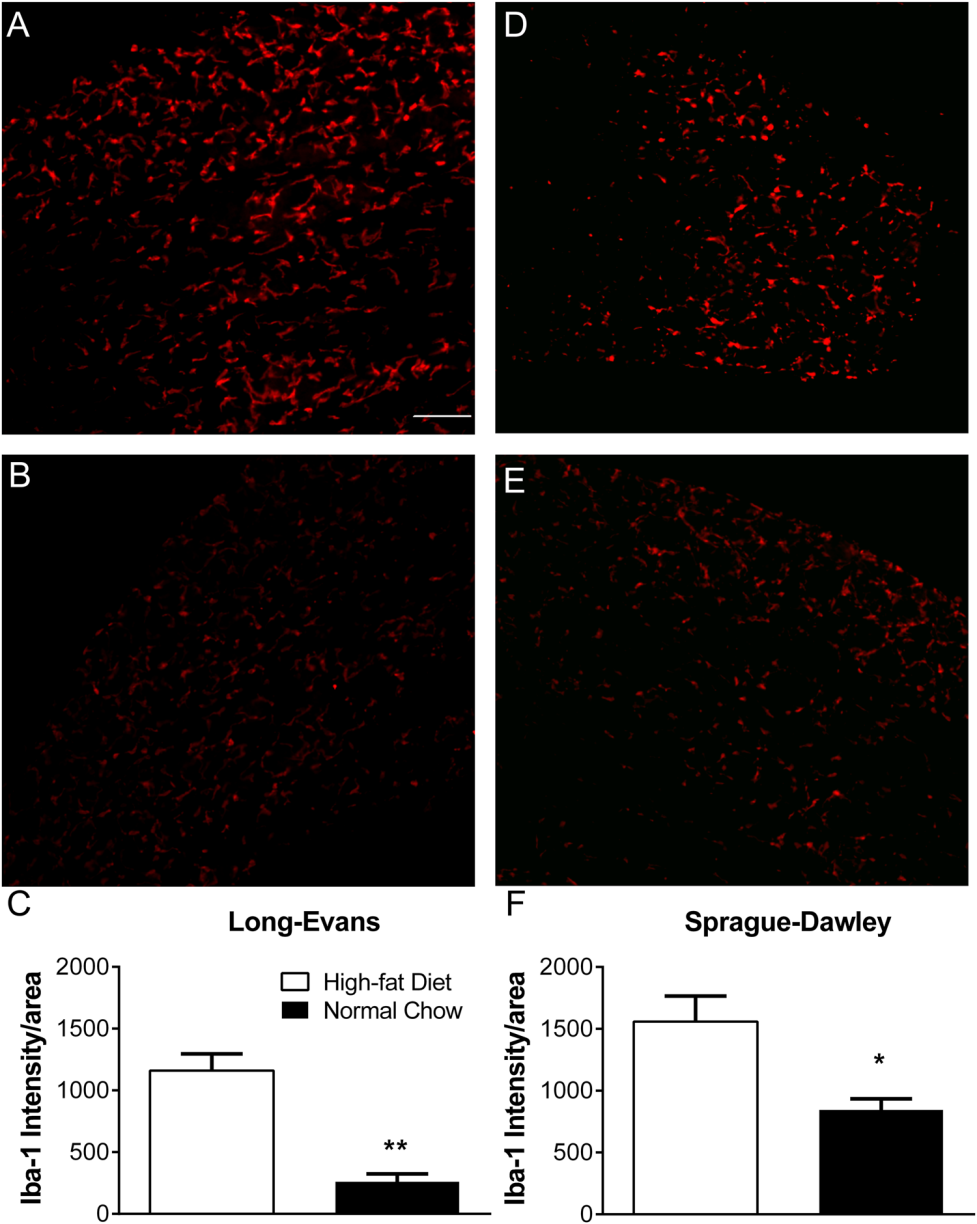
High-fat diet increases macrophage density in the lumbar DRG. Sample micrographs of Iba-1 labelling in cross sections of DRG from rats fed a high-fat diet for 6 weeks (**A**, Long-Evans; **D**, Sprague-Dawley) or maintained on normal chow (**B**, Long-Evans; **E**, Sprague-Dawley). No pain model was implemented. Scale bar 100 µm. Summary data of normalized intensity is shown in **C** (Long-Evans) and **F** (Sprague-Dawley). N = 4 male Sprague-Dawley or 5 male Long-Evans rats per group, with 17–44 sections measured per animal. *p < 0.05; **p < 0.01; significantly different from high-fat group (Mann-Whitney test).

One mechanism by which high-fat diet could enhance the response to pain models is through increasing baseline inflammatory processes. As an initial investigation into this class of mechanisms, we measured macrophage density in the DRG in rats maintained on the high-fat diet for 6 weeks in the absence of any pain models. As shown in Fig. 8, the summed intensity of the Iba-1 immunolabeling was over 4-fold higher in male Long-Evans rats fed the high-fat diet. Macrophage density was also increased in female Long-Evans rats fed the high-fat diet, in which the summed intensity was 1.8 ± 0.26 fold higher than in rats fed normal chow (p = 0.04, Mann-Whitney test, n = 6 per group). In contrast, no differences in Iba-1 immunolabeling were observed in lumbar spinal cord sections obtained from the same animals (average normalized intensity values = 353 ± 69 in the high-fat diet group and 348 ± 66 in the normal chow group, p = 1.0, Mann-Whitney test.) Increased Iba-1 immunolabeling was also observed in DRGs from Sprague-Dawley rats, though the increase was more modest (1.8 fold; Fig. 8F). We also examined GFAP, a marker for satellite glia activation in the DRG^44^. There was very little GFAP labeling in the DRG regardless of diet (data not shown), as expected for DRG taken from animals in the absence of a pain model. GFAP labeling was largely absent in DRGs from animals maintained on either a high-fat diet or normal chow for six weeks, in which no pain model had been implemented (n = 4 male Long-Evans rats per group).

## Discussion

In this study, we used milder versions of two different pain models, local DRG inflammation and CFA paw injection, to demonstrate that a high-fat diet (40% calories from fat, primarily butter fat, for 6 weeks prior to implementing the models) markedly enhanced pain behaviors. Measured behaviors that were affected included static and dynamic mechanical allodynia and cold allodynia, as well as guarding behavior, a measure of spontaneous pain^38^. In the peripheral inflammation model (paw injection of CFA), high-fat diet also exacerbated paw swelling. The relatively large effects of diet on cold allodynia contrast with our recent study^34^ showing that localized sympathectomy markedly reduced DRG inflammation and mechanical pain behaviors but had much smaller effects on cold allodynia. This suggests that diet-induced modulation of the sympathetic system may not be the major mechanism underlying the effect of high-fat diet. However, it should be noted that several studies show that the sympathetic system can be activated in obesity, and in particular may play a role in obesity-induced hypertension^45–47^. In contrast, a high-fat diet impaired sympathetic activation of brown fat in Sprague Dawley rats; like the effects on pain in our study, this effect did not depend on the animals becoming obese^48^.

The paradigm of diet-induced obesity that we used was originally characterized in Long-Evans rats^31^. However, our previous work with these pain models had been carried out in the Sprague-Dawley strain. These circumstances led us to test the effects of diet on pain in both strains. We found that the Sprague-Dawley rats did not gain weight after 6 weeks high-fat diet exposure, and did not develop the metabolic hallmarks of obesity that were seen in the Long-Evans rats, such as increased percent body fat and elevated plasma leptin, insulin, and leptin/adiponectin ratio. Hence the enhanced pain responses we observed after feeding the high-fat diet cannot be readily attributed to increased circulating insulin and leptin, nor to the mechanical strain due to increased skeletal weight. Moreover, the pain-modulatory effects of the high-fat diet apparently take some time to develop, since we could not fully mimic the pain phenotype by providing this diet for only one week prior to implementing the DRG inflammation model. Similar to this finding, a study in mice genetically engineered to respond to a high-fat diet with elevated cholesterol and blood lipids, but not increased body weight, found that the observed effects of diet on T cell function required many weeks to develop even though blood cholesterol and lipids were markedly elevated within one week^49^. Collectively, this suggests that high-fat diet may alter immune system function through a process that takes several weeks to develop, and that can sometimes be dissociated from any effects on body weight and adiposity.

Importantly, type I inflammation contributes to pain behaviors in both of the pain models used in the present work, suggesting that high-fat diet may mediate its effects by promoting type 1 inflammatory responses. For example, CFA-induced paw inflammation (which strongly stimulates a type 1 response) was augmented by high-fat diet. Diet-induced obese rats also had greater paw edema and arthritis scores in a study using a higher dose of CFA that was sufficient to induce monoarthritis of the ankle^28^. In that study, the magnitude of the high-fat diet effect appeared to be more modest than observed in the present work, possibly because we minimized assay ceiling effects by using a milder version of the CFA model. Similarly, Totsch *et al*.^29^ observed prolongation of pain behaviors in the CFA model in mice fed a Total Western Diet, rather than an increase in the maximum responses; differences between that study and this one may simply reflect our use of milder models that allow room for the maximum response to increase. Other studies in both humans and rodents also provide evidence for increased inflammatory responses, including studies showing that obese adipose tissue contains higher numbers of macrophages that are more M1 polarized (see Introduction), as well as studies showing systemic increases in pro-inflammatory cytokines (e.g., IL-6, IL-1β, MCP-1) in obese individuals (see Introduction). However, the latter effect is not observed in all studies; in some studies, including the present work, elevation of these systemic cytokines is not detected (e.g. ref. 49). Human studies indicate that systemic elevation of these markers may depend on the degree of metabolic dysregulation and/or the specific deposition of adipose tissue (e.g., visceral vs. subcutaneous) rather than on total adiposity per se^50, 51^.

Another class of mechanisms that may mediate the pain-promoting effects of high-fat diet are direct effects in the nervous system. Supporting a role for such processes is our observation that the number of macrophages in the DRG increased in animals of both strains on the high-fat diet, prior to any pain model being implemented. Macrophage infiltration of the DRG is observed in the local DRG inflammation model^30^, as well as in many other pain models, and macrophages have been implicated in the resulting pain behaviors^52^. The importance of direct effects in nervous tissue is also supported by a recent study^27^ of the role of peroxisome proliferator-activated receptor-α (PPARα) in pain behaviors in rats with high-fat diet-induced obesity. This work demonstrated reductions in behavioral responses to peripheral inflammation after i.t. injection of a drug that reversed obesity-induced PPARα downregulation in spinal cord, even though the treatment did not affect metabolic parameters. This widely-expressed anti-inflammatory receptor, which can directly sense fatty acids and lipid-derived substrates in many tissues, is downregulated by both obesity^53^ and local DRG inflammation^54^.

Systemic and neuron-specific effects of high-fat diets may interact to give the pain phenotypes we observed in the non-obese Sprague-Dawley rats. For example, loss of activation of PPAR receptors could result from changes in fat-derived substances in the blood, or to changes in the liver, even in the absence of increased adipose tissue, with the primary effects on pain resulting from PPAR receptors in neurons rather than other tissues (as in the above-cited study). It will be of interest to determine whether the high-fat diet can promote fatty livers despite not affecting whole body adiposity. Our finding that high-fat diet alone increases macrophage density in the DRG has parallels in a recent study of chemotherapy-induced pain^55^ which showed that development of pain required a normal gut microbiome, and that chemotherapy increased pro-inflammatory cytokines in the DRG but not plasma or spinal cord. In that study, macrophages were shown to play a key role in mediating the effects of chemotherapy and of changed gut microbiomes, and pain was associated with increased macrophage density in the DRG. Interestingly, changes in gut permeability and microbiome have been proposed to be one mediator of high-fat diet-induced obesity^56, 57^, and differences in microbiome have been proposed to account for some of the differences between rat strains in susceptibility to high-fat diets^41^. It will be of interest to examine these possible mechanisms by which high-fat diet per se might influence DRG neurons and pain even without increased body fat.

In summary, this study provides evidence that a high-fat diet can increase pain behaviors in two different rat pain models that involve local inflammation, even in the absence of body weight gain and some of the other metabolic changes associated with obesity. The ability of high-fat diet to modify pain responsiveness even without concurrent obesity may be a previously under-appreciated contributor to pain conditions in humans. Diet (independent from body weight) may also represent a critical factor for consideration in human pain studies.

## Electronic supplementary material

Supplementary Information
