# Supplementary material for: High-fat diet increases pain behaviors in rats with or without obesity
Source: Sci Rep. 2017 Sep 4;7:10350. doi: 10.1038/s41598-017-10458-z (PMC5583349; doi:10.1038/s41598-017-10458-z)
Supplement: Supplementary file 1 — Supplementary Information [file 41598_2017_10458_MOESM1_ESM.pdf]

Supplemental Figures and Table for:

High-fat diet increases pain behaviors in rats with or without obesity

Zongbin Song, Wenrui Xie, Sisi Chen, Judith A. Strong, Mason S. Print, Joy I. Wang, Aleeya F. Shareef, Yvonne M. Ulrich-Lai, and Jun-Ming Zhang.

Supplemental Figure 1

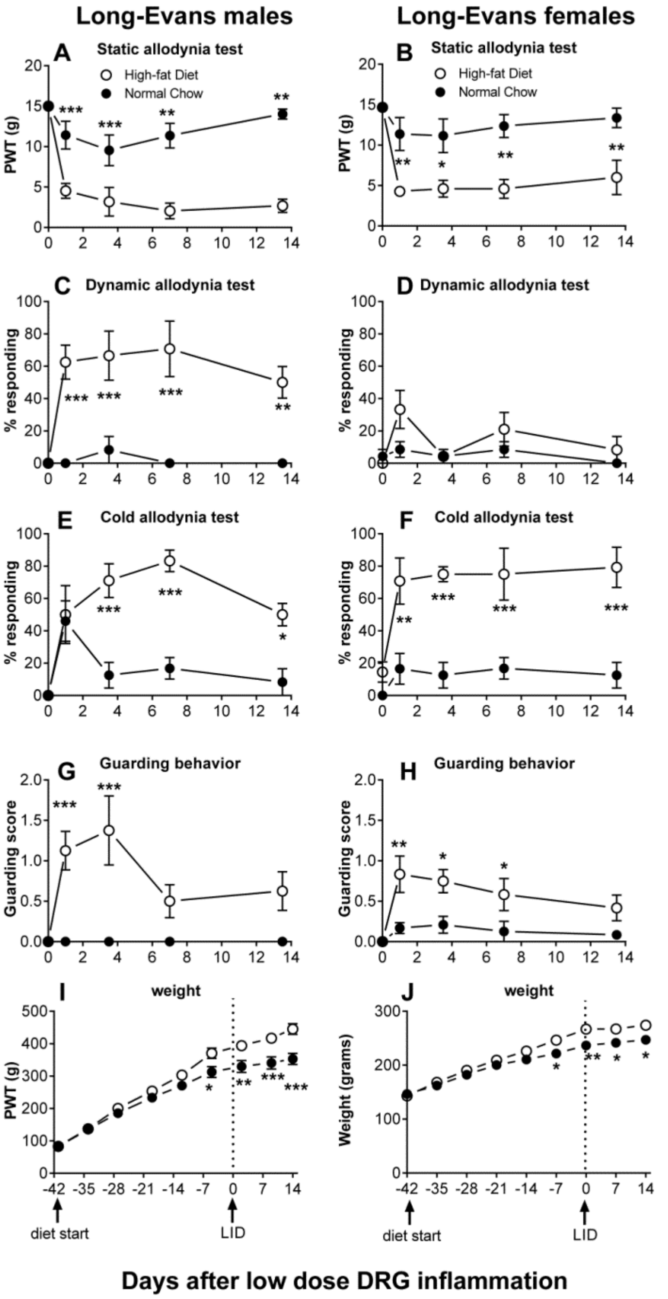

Supplemental figure 1. The low dose DRG inflammation model causes marked pain behaviors in Long-Evans male or female rats maintained on a high-fat diet. The low dose DRG inflammation model was implemented on day 0, after animals had been maintained on the indicated diet for 6 weeks. Baseline behaviors were measured twice just before the pain model was implemented (average plotted on day 0). The high-fat diet group, but not the normal chow group, responded to the DRG inflammation with a marked decrease in von Frey threshold in both males (A) and females (B). An increase in dynamic mechanical allodynia was significant in males (C) but did not reach significance in females (D). Cold allodynia was significantly elevated on most days in male (E) and females (F), as were guarding behavior scores (G, males; H; females). N = 4 Long-Evans rats of the indicated sex per group. The same data are shown in figure 4 with data from males and females combined. I, male, and J, female animals fed a high fat diet had significantly higher weights starting after 5 weeks on the diet . *, p<0.05; **, p<0.01; ***, p<0.001; significant difference between groups at indicated time points (2-way repeated measure ANOVA with Sidak's multiple comparison posttest); $F_{(1,6)}$ = 26.2 (A), 16.9 (B), 23.8 (C), 3.2 (D), 38.9 (E), 38.3 (F), 42.8 (G), 12.34 (H), 7.3 (I), and 5.5 (J).

Supplemental Figure 2

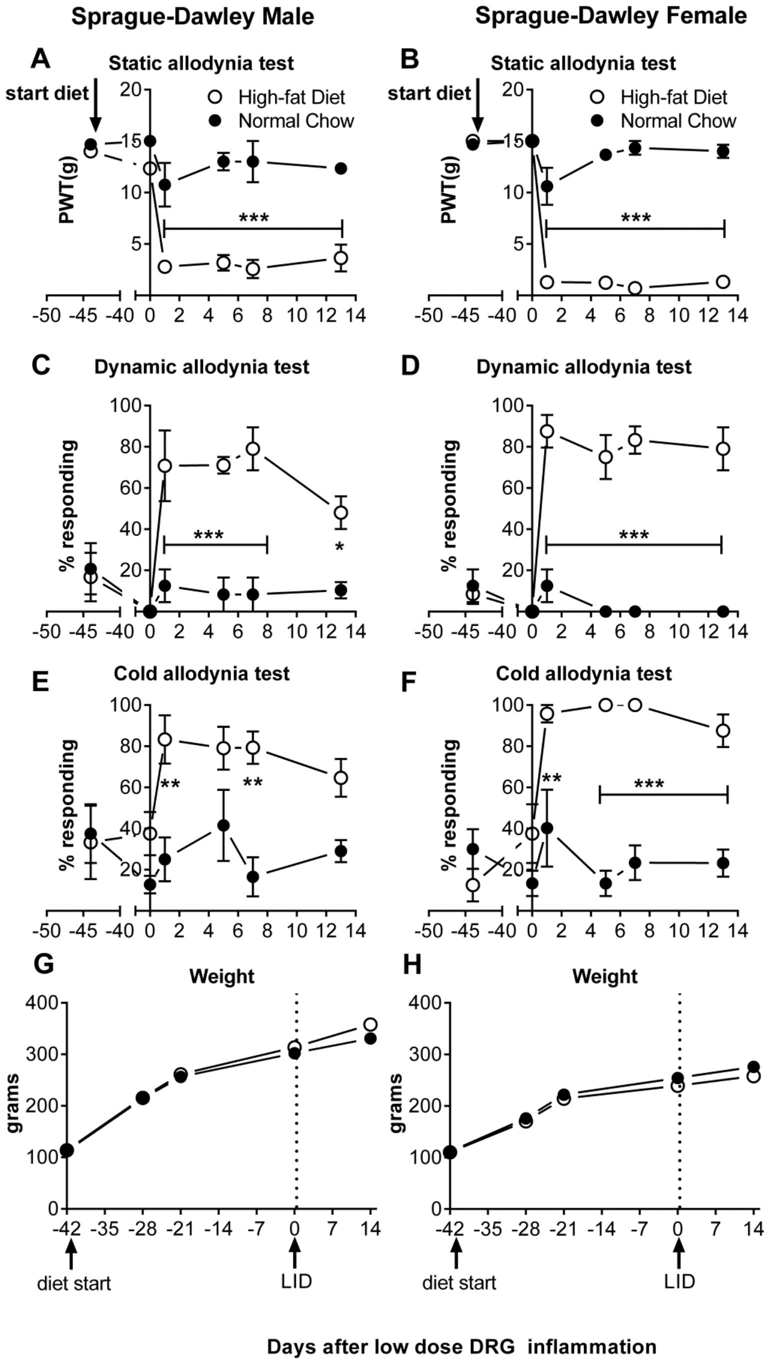

Supplemental Figure 2. Effects of the low dose DRG inflammation model in Sprague-Dawley male or female rats maintained on a high-fat diet. Baseline behaviors were measured just before animals were started on the diet (high-fat diet group; control animals continued on the standard low fat chow diet) (plotted on POD -44). Behaviors were measured again after 6 weeks on the diet and just prior to surgery for DRG inflammation with low dose zymosan (POD 0). The high-fat diet group, but not the normal (low fat) chow group, responded to the zymosan (2 µg) injection into the DRG with a marked decrease in von Frey threshold in both males (A) and females (B), along with an increase in dynamic mechanical allodynia in males (C) and females (D). Cold allodynia increases reached significance on some days in males (E) and females (F). N = 4 Sprague-Dawley rats of the indicated sex per group. The same data are shown in figure 5 with data from males and females combined. Diet did not significantly affect weight in males (G) or females (H). **, p<0.01; ***, p<0.001; significant difference between groups at indicated time points (2-way repeated measure ANOVA with Sidak’s multiple comparison posttest); $F_{(1,6)}$ = 134.6 (A), 320.6 (B), 67.1 (C), 117.8 (D), 27.8 (E), 34.5 (F), 1.18 (G), and 2.41 (H).

Supplemental Figure 3

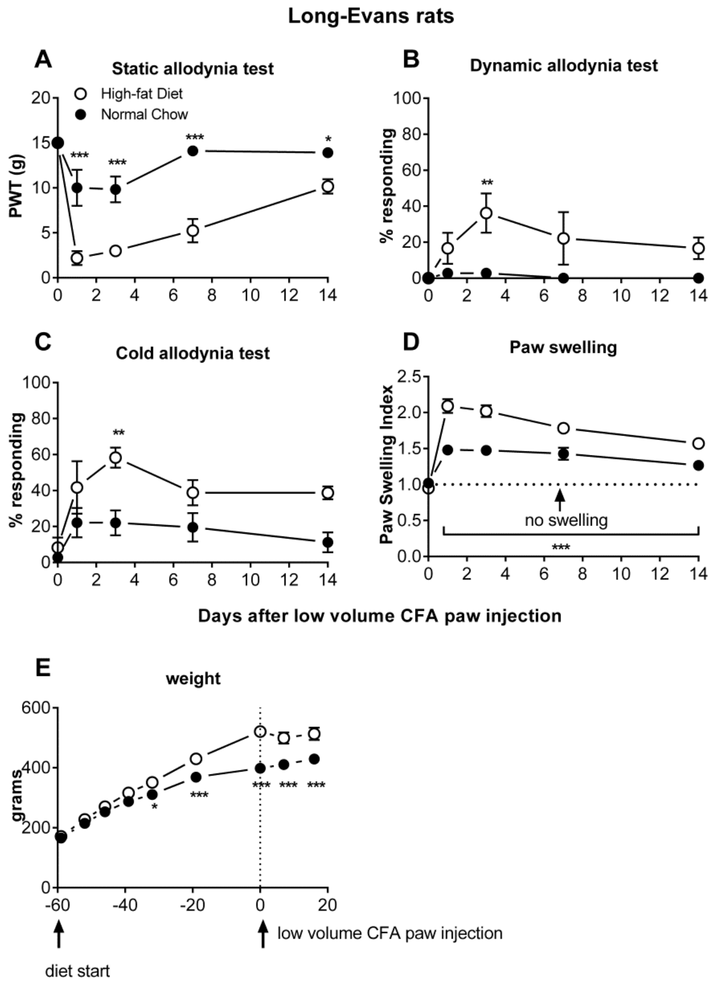

Supplemental Figure 3. Pain behaviors in a milder paw inflammation model are exacerbated in Long-Evans rats that are maintained on a high-fat diet. Animals were maintained on high-fat diet or normal chow starting 6 weeks prior to implementing the pain model. Baseline behaviors were measured just prior to injection of the ipsilateral paw with 10 µL of 50% Complete Freund's Adjuvant (CFA) on postoperative day (POD) 0 (volume reduced from the 50 µL that is typically used). (A) The high-fat diet group showed larger decreases in von Frey threshold at most post-CFA time points relative to the normal chow group. Differences between the groups in in mechanical (B) and cold (C) allodynia reached significance only on post-CFA day 3. Paw swelling index (area of ipsilateral paw divided by area of contralateral paw; index of 1 = no swelling) was also significantly greater on all post-CFA days in animals eating a high-fat diet. (E). Higher weight in rats consuming the high-fat diet was significant within 4 weeks on diet. N = 6 male Long-Evans rats per group. *, p<0.05; **, p<0.01; ***, p<0.001; significant difference between groups at indicated time points (2-way repeated measure ANOVA with Sidak's multiple comparison posttest, $F_{(1, 10)}$ = 40.1, von Frey data; 13.4, mechanical allodynia data; 14.6, acetone data; 25.3, paw swelling data; 25.2, weight data).

Supplemental Figure 4

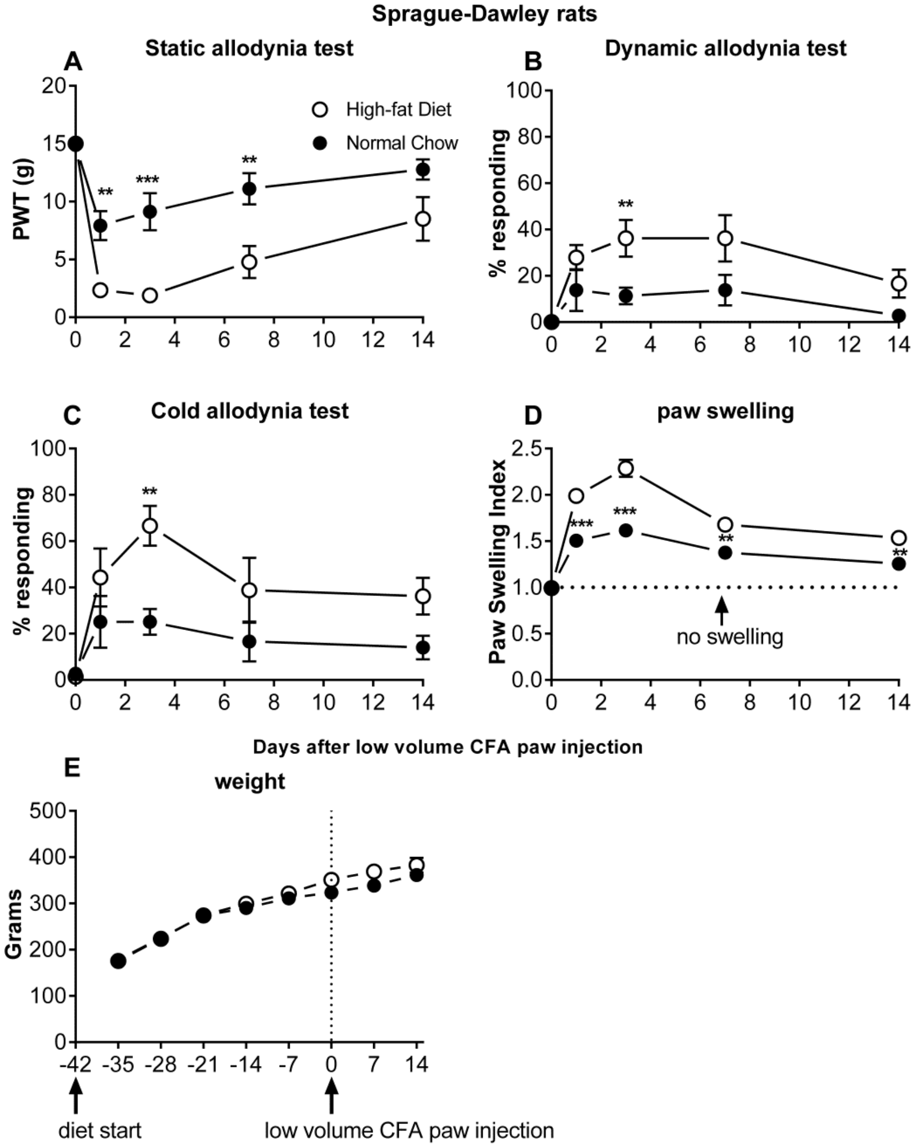

Supplemental Figure 4. Pain behaviors in a milder paw inflammation model are exacerbated in Sprague Dawley rats that are maintained on a high-fat diet. Animals were maintained on high-fat diet or normal chow starting 6 weeks prior to implementing the pain model. Baseline behaviors were measured just prior to injection of the ipsilateral paw with 10 µL of 50% Complete Freund's Adjuvant (CFA) on postoperative day (POD) 0 (volume reduced from the 50 µL that is typically used). (A) The high-fat diet group showed larger decreases in von Frey threshold at all post-CFA time points relative to the normal chow group. Differences between the groups in in mechanical (B) and cold (C) allodynia reached significance only on post-CFA day 3. Paw swelling index (area of ipsilateral paw divided by area of contralateral paw; index of 1 = no swelling) was also significantly greater in animals eating a high-fat diet on all post-CFA days. Weights did not differ significantly between the two groups (E). N = 6 male Sprague-Dawley rats per group. *, p<0.05; **, p<0.01; ***, p<0.001; significant difference between groups at indicated time points (2-way repeated measure ANOVA with Sidak's multiple comparison posttest, $F_{(1, 10)}$ = 28.3, von Frey data; 6.6, mechanical allodynia data; 5.9, acetone data; 48.2, paw swelling data; 0.89, weight data).

Supplemental figure 5.

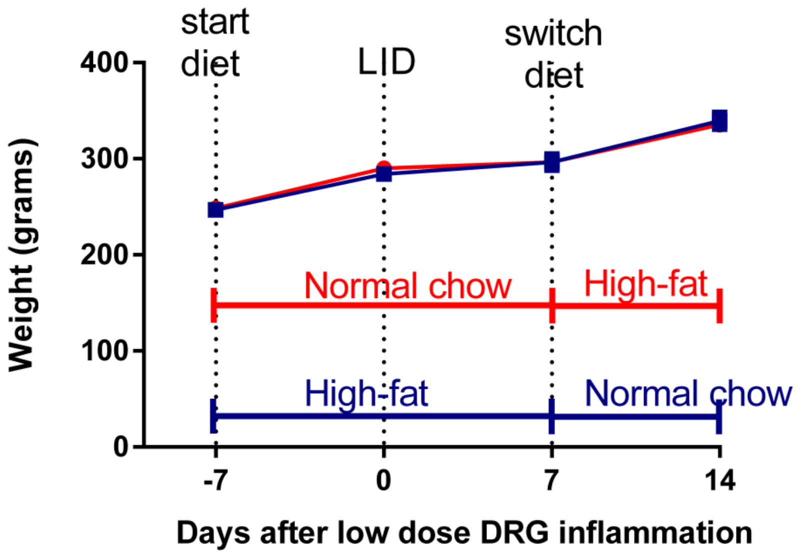

Supplemental Figure 5. Short-term diet changes did not affect weight in Sprague-Dawley rats. Weight data from animals for the experiment shown in Figure 6. Rats were maintained on normal chow until reaching a weight of ~280 grams, to have age and weight comparable to animals in the other experiments at the time the pain model was implemented. Low dose DRG inflammation ("LID") was implemented at time 0. Seven days prior, one group (blue symbols) was switched to high-fat diet while the other group (red symbols) continued on normal chow. 1 week after the low dose DRG inflammation the diets of the two groups were switched. There were no significant differences in weight between the two groups at any time point (2-way repeated measure ANOVA with Sidak's multiple comparison posttest, $F_{(1, 14)}$ = 0.0099, p = 0.92).

Supplemental Table 1

$F_{(1, 28)}$ values from 2-way ANOVA analysis of effect of strain and diet on adipokine and cytokine levels presented in Figure 7.

	Diet effect	Strain effect	Interaction
Adiponectin	2.4	1.77	0.00036
Insulin	10.89**	38.07***	7.55*
Leptin	27.77***	31.34***	5.09*
L/A ratio	15.99***	16.08***	1.5
MCP-1	0.157	3.12	0.042
IL-1B	0.043	9.32**	1.09

*, $p<0.05$; **, $p<0.01$; ***, $p<0.001$, significant effect.
